# Synchronous Parathyroid Carcinoma and Noninvasive Follicular Thyroid Neoplasm With Papillary-Like Nuclear Features

**DOI:** 10.7759/cureus.24006

**Published:** 2022-04-10

**Authors:** Ahmed Alajaimi, Noor Altooq, Nisha Chandran, Yaser Alderazi

**Affiliations:** 1 College of Medicine & Medical Science, Arabian Gulf University, Manama, BHR; 2 Department of Pathology, Salmaniya Medical Complex, Manama, BHR; 3 Department of General Surgery, Salmaniya Medical Complex, Manama, BHR

**Keywords:** niftp, total thyroidectomy, primary hyperparathyroidism, thyroid carcinoma, parathyroid carcinoma

## Abstract

Parathyroid carcinoma is a rare cause of primary hyperparathyroidism. Compared to parathyroid adenoma, parathyroid cancer is more likely to be associated with marked levels of serum parathyroid hormone (PTH) and hypercalcemia with severe clinical manifestations. Noninvasive follicular thyroid neoplasm with papillary-like nuclear features (NIFTP) is a rare variant of papillary thyroid cancer. Here, we report the case of a middle-aged woman who presented with multiple fractures and neck swelling. Neck ultrasound and fine-needle aspiration cytology revealed a provisional diagnosis of thyroid carcinoma. Parathyroid and bone scan were performed because of primary hyperparathyroidism and hypercalcemia and established the diagnosis of hyperfunctioning right-sided parathyroid tumor. Right parathyroidectomy and total thyroidectomy were performed, and the histopathological report confirmed the diagnosis of parathyroid carcinoma and NIFTP. The synchronic coexistence between parathyroid cancer and thyroid neoplasms is an extremely rare condition that prompted us to report this case.

## Introduction

Parathyroid cancer is one of the rare endocrine malignancies accounting for 0.5-5% of cases of primary hyperparathyroidism [1]. Although benign parathyroid disease and parathyroid cancer can share the same clinical presentation, some features increase the clinical suspicion of parathyroid cancer [2]. Compared to parathyroid adenoma, parathyroid cancer is more likely to present with a larger tumor, marked levels of serum parathyroid hormone (PTH), malignant hypercalcemia, nephrolithiasis, and bone manifestations [3].

Despite the rarity of the incidence of parathyroid cancer among all other types of malignant tumors, the coexistence between thyroid and parathyroid malignancies is considered an extremely rare finding. Noninvasive follicular thyroid neoplasm with papillary-like nuclear features (NIFTP) is another rare entity of thyroid carcinoma [4]. NIFTP is an encapsulated noninvasive follicular variant of papillary thyroid cancer that is characterized by low malignancy potential and low risk of recurrence and spread [5]. This is clinically manifested by its low tendency for extrathyroidal invasion or lymph node metastasis [5]. The preoperative diagnosis of concurrent thyroid and parathyroid neoplasms poses several challenges and requires the constellation of thorough clinical, radiologic, and pathologic investigations.

To the best of our knowledge, our patient is the first documented case of synchronous parathyroid carcinoma and NIFTP. The inadequacy in implementing proper diagnostic workup and management guidelines for such conditions has prompted us to document this case.

## Case presentation

A 51-year-old Filipino female presented to the general surgery department in 2018 with neck swelling. A detailed history demonstrated that the patient was asymptomatic. There was no history of palpitations, heat or cold intolerance, constipation, diarrhea, fatigue, menstrual abnormalities, hair loss, dysphagia, difficulty breathing, or hoarseness of the voice. A physical examination showed that the patient had an anterior neck swelling that was hard, fixed, nontender, and 5 × 6 cm in size with no palpable lymph nodes. Swallowing was normal, and Pemberton’s sign was negative. Thyroid function tests were within the normal levels.

An ultrasound of the neck revealed that both lobes of the thyroid gland were enlarged, with the presence of a large nodule in each lobe. The right lobe showed a moderately suspicious lesion measuring 3.2 × 5 × 5 cm in size with increased vascularity, macrocalcification, and a Thyroid Imaging Reporting and Data System (TI-RADS) score of 4. The left lobe showed a mildly suspicious lesion measuring 2.9 × 1.9 × 3.9 cm with peripheral vascularity, no evidence of internal calcification, and a TI-RADS score of 3. There were no significant enlarged lymph nodes on either side of the neck. Ultrasound-guided fine-needle aspiration cytology (FNAC) showed sheets and clusters of follicular cells infiltrated with occasional cells that showed moderate pleomorphism and large naked nuclei in the right lobe of the thyroid gland. The left lobe showed follicular cells arranged predominantly in small clusters and microfollicles with epithelial cells showing overlapping nuclei, a few intranuclear grooves, and rare intranuclear cytoplasmic inclusions. The nodule in the right lobe was diagnosed as atypia of undetermined significance (category III) according to the Bethesda system for reporting thyroid cytopathology, whereas the nodule in the left lobe had features suspicious of papillary thyroid carcinoma which was classified as category V accordingly. The patient was scheduled for a total thyroidectomy but she missed all her appointments and was lost to follow-up.

On November 19, 2021, the patient was admitted to the orthopedic department with a left subtrochanteric femur fracture, right undisplaced tibia fracture, and failure of dynamic hip screw implant of the right femur because of previous right subtrochanteric femur fracture which occurred one month prior. The patient was noted to have a large neck mass by the orthopedic department which mandated consultation from the general surgery department.

Upon second surgical evaluation, the patient was found to have chronic shortness of breath that worsened on both rest and exertion, constipation, and generalized body pain. Examination showed hard diffuse neck swelling more prominent on the right side, associated with tracheal deviation. Pemberton’s sign was negative. Lab investigations showed serum hypercalcemia and increased serum levels of PTH and 25-OH vitamin D (Table 1). No renal diseases were found, and a diagnosis of primary hyperparathyroidism was made.

**Table 1:** Summary of laboratory findings before surgical intervention.

Laboratory investigation	Result	Unit	Reference range
White blood cell count	5.41	×10^9^/L	3.6–9.6
Hemoglobin	12.7	g/dL	12.0–14.5
Platelets	289	×10^9^/L	150.0–400.0
Thyroid-stimulating hormone	0.3	mIU/L	0.25–5.0
Free thyroxine	7.6	pmol/L	6.0–24.5
Anti-thyroglobulin	0	IU/mL	<100
Thyroglobulin	4	μg/L	3.0–42.0
Parathyroid hormone	220	pmol/L	1.96–9.33
Calcium	3.65	mmol/L	2.15–2.50
Inorganic phosphorus	0.5	mmol/L	0.81–1.45
Magnesium	0.9	mmol/L	0.74–1.00
25-OH vitamin D	192	nmol/L	50–125
Cancer antigen 125	8.1	U/mL	<35
Cancer antigen 19-9	2.5	U/mL	<37
Carcinoembryonic antigen	3.5	μg/L	1.8–3.5

**Figure 1:**
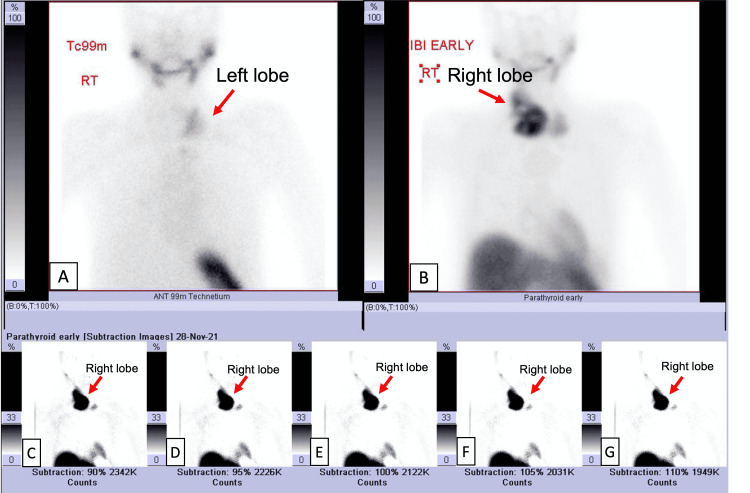
Parathyroid scan. (A) Initial images of the thyroid gland using Tc-pertechnetate shows an irregular accumulation of the tracer in the region of the left lobe of the thyroid (arrow). (B) The To-MIBI parathyroid images show a large irregular area of ​​accumulation of the tracer in the region of the right thyroid lobe (arrow). (C-G) Accumulation of the tracer in the right thyroid lobe remains the same on the delayed images (arrow). MIBI: methoxyisobutyl isonitrile

**Figure 2:**
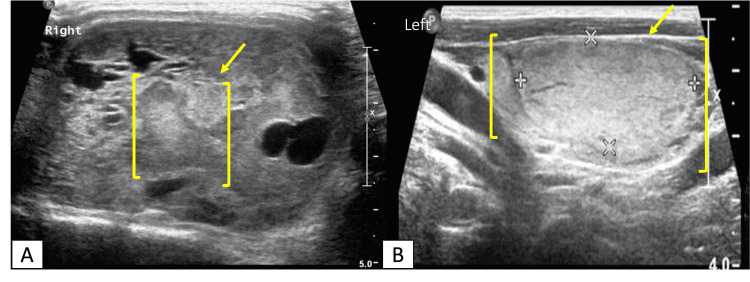
Neck ultrasound. (A) Right lobe lesion (TI-RADS 4): moderately suspicious lesion with size more than 1.5 cm. (B) Left lobe lesion (TI-RADS 3): mildly suspicious lesion with size more than 2.5 cm. TI-RADS: Thyroid Imaging Reporting and Data System

A chest X-ray showed displacement of the trachea on the right side. A parathyroid scan revealed a hyperfunctioning parathyroid tumor in the right lobe (Figure 1). An ultrasound of the neck was obtained and compared to the previous one done in 2018 (Figure 2). There was mild compression and displacement of the trachea on the right side but there was no mediastinal or retrosternal extension and no change in the radiologic characteristics of the right and left nodules. Moreover, there was no enlargement of the lymph nodes. A bone scan was performed for assessment of metastatic or lytic lesions of parathyroid carcinoma. 740 MBq of Tc-99m MDP was intravenously administered. Multiple pathologic fractures involving both lower limbs and pelvic bones showed mildly increased uptake. There was also increased uptake involving the axial and appendicular skeleton and soft tissue uptake (lungs, stomach, and kidneys) which was likely due to hypercalcemia and hyperparathyroidism.

The patient underwent total thyroidectomy and right parathyroidectomy in view of suspicious lesions and compressive symptoms. On resection, the large nodular mass on the right side measured 7 × 6 × 3.5 cm and showed an encapsulated lesion with gray-white granular and necrotic areas. A well-circumscribed nodule in the left lobe of the thyroid gland measured 3.5 × 2.5 × 2.3 cm and showed gray tan areas with focal hemorrhage on serial sectioning.

**Figure 3:**
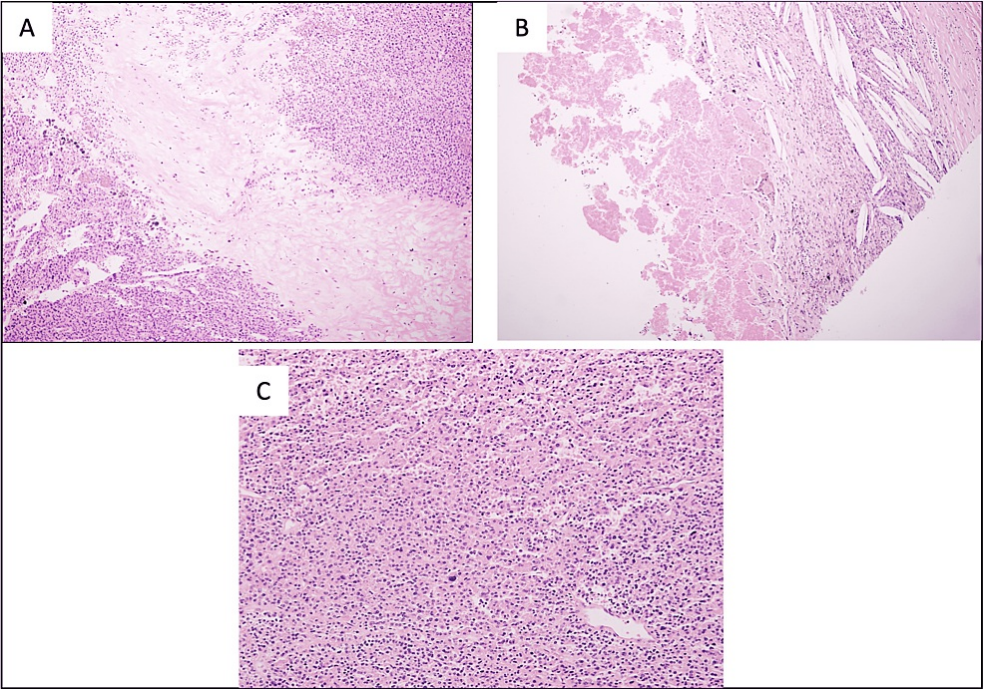
Histopathological features of the parathyroid lesion. (A) Parathyroid lesion with fibrous capsule, many broad fibrous septa are dividing the tumor into multiple lobules (H&E, 10×). (B) Extensive areas of necrosis, hemosiderin-laden macrophages, and cholesterol clefts are noted within the mass (H&E, 20×). (C) Tumor lobules are composed of nests of clear cells with an admixture of eosinophilic cells (H&E, 20×). H&E: hematoxylin and eosin

**Figure 4:**
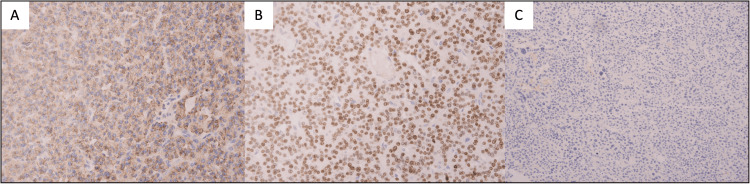
Immunohistological features of the parathyroid lesion. The tumor cells are positive for chromogranin and GATA3 (strong and diffuse) and negative for all thyroid markers such as thyroglobulin, TTF-1, and PAX-8 (H&E, 20×). GATA3: GATA binding protein; TTF-1: thyroid transcription factor 1; PAX-8: paired-box gene 8; H&E: hematoxylin and eosin

The right-sided nodule showed histological features such as clusters of clear and eosinophilic cells with neuroendocrine nuclear features, broad fibrous septa, and immunohistochemical features such as lack of thyroid markers along with positive CAM 5.2, chromogranin, and GATA binding protein 3 (GATA3) which support the parathyroid origin of the lesion. Another supporting feature is the clinical presentation of increased calcium and serum PTH levels, as well as multiple bone fractures. Additionally, serum PTH and calcium values decreased after excision of the neoplasm (Table 2). Tumor cells were negative for thyroid transcription factor 1 (TTF-1), thyroglobulin, and paired-box gene 8 (PAX-8). Calcitonin, carcinoembryonic antigen (CEA), pancytokeratin, S-100, synaptophysin, and CD56 were also negative. The parathyroid tumor showed atypical features such as broad fibrous septa, atypia, and necrosis. Rare foci suspicious of vascular invasion were noted. Of note, the right-sided tumor showed adhesion to the muscles and the esophagus on the right side which further supported the diagnosis of parathyroid malignancy (Figures 3, 4).

**Table 2:** Summary of laboratory findings after surgical intervention.

Laboratory investigation	Result	Unit	Reference range
White blood cell count	4.91	×10^9^/L	3.6–9.6
Hemoglobin	9.4	g/dL	12.0–14.5
Platelets	277	×10^9^/L	150.0–400.0
Thyroid-stimulating hormone	56.71	mIU/L	0.25–5.0
Parathyroid hormone	4.90	pmol/L	1.96–9.33
Calcium	1.95	mmol/L	2.15–2.50
Inorganic phosphorus	0.8	mmol/L	0.81–1.45
Magnesium	0.8	mmol/L	0.74–1.00
25-OH vitamin D	71	nmol/L	50–125

**Figure 5:**
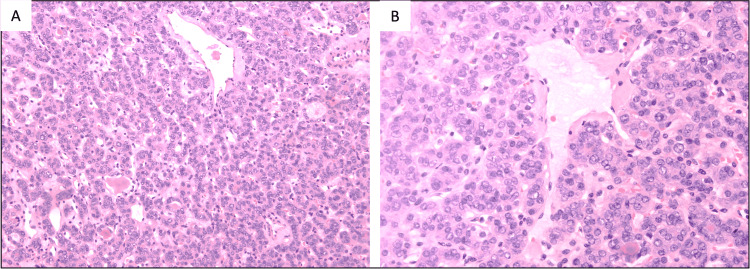
Histopathological features of the thyroid lesion. (A) The thyroid nodule is composed of micro and macrofollicles lined by follicular epithelial cells showing patchy nuclear clearing, overlapping, nuclear membrane irregularities, and pleomorphism (H&E, 10×). (B) No definitive intranuclear inclusions noted in the section studied. No vascular or capsular invasion seen. No papilla, necrosis, or solid areas noted (H&E, 10×). H&E: hematoxylin and eosin

The left-sided thyroid nodule showed patchy nuclear features of papillary thyroid carcinoma and exclusively follicular patterned lesion. No capsular or vascular invasion was noted. These features collectively supported the diagnosis of NIFTP (Figure 5).

## Discussion

We have demonstrated the unique constellation of parathyroid carcinoma and NIFTP in a case manifesting with symptoms of primary hyperparathyroidism and hypercalcemia.

Parathyroid cancer can occur sporadically or as part of other diseases such as multiple endocrine neoplasia (MEN) 1 or MEN 2, familial hyperparathyroidism, and hyperparathyroidism-jaw tumor syndrome (HPT-JT) [6]. Mutation of the CDC73 (also called HRPT2) tumor suppressor gene plays a central role in the pathogenesis of parathyroid cancer associated with jaw tumor syndrome and can be the cause of marked hypercalcemia and its clinical manifestations [7].

The patient in this case presented with marked levels of serum PTH manifesting with hypercalcemia and multiple fractures within a short time interval. The effect of parathyroid carcinoma on the serum levels of PTH is more significant than with adenomas. It dramatically affects the renal function, gastrointestinal system, and the metabolism of bones as it can increase the level of serum PTH more than any other cause of primary hyperparathyroidism [8,9]. In a case report published in 2017, a 68-year-old female diagnosed with parathyroid carcinoma was found to have a PTH level that was 18 times higher than the upper normal limit of the hormone [10]. This finding justifies the correlation between serum levels of PTH and the severity of symptomatology.

The initial presentation of the patient, combined with the ultrasound and FNAC results, led to the establishment of a provisional diagnosis of a thyroid tumor, and the patient was accordingly scheduled for total thyroidectomy. The late clinical manifestations of primary hyperparathyroidism necessitated the performance of the parathyroid scan. This confirms the significance of full laboratory and radiologic investigations to rule out any concurrent parathyroid lesion.

For the establishment of a definitive diagnosis of suspicious nodules, a thorough histopathological examination is mandatory. The detection of capsular and vascular invasion, trabecular pattern, and mitotic figures along with the use of immunohistological stains (chromogranin, GATA3, CAM 5.2, TTF1, thyroglobulin, PTH, parafibromin) is necessary for the identification of the origin of the lesion [11].

NIFTP is an encapsulated noninvasive follicular variant of papillary thyroid cancer which shares the RAS mutations with follicular thyroid neoplasms [12]. It is characterized by its low tendency for metastasis and rarely progresses from its benign form to malignancy [5]. Capsular or vascular invasion, lymph node metastasis, and extrathyroidal invasion are what differentiate the encapsulated follicular variant of papillary thyroid cancer from NIFTP. Thus, the management of a thyroid neoplasm that presents as NIFTP differs from other types of neoplasms. Studies show that lobectomy is the best management approach for NIFTP and is favored over total thyroidectomy. The significance of classifying NIFTP in this new category is to prevent aggressive surgical management as it has a good prognosis [5,13].

The preoperative diagnosis of a synchronous thyroid tumor and parathyroid cancer can be quite difficult as there are no clear guidelines of management for these conditions. Intraoperatively, the right-sided nodule had adhesions with the adjacent muscles and the esophagus on the right side in this case. In this context, the resection of the parathyroid tumor and the ipsilateral lobe of the thyroid gland is necessary in case there is an intraoperative suspicion of parathyroid carcinoma. Even though NIFTP can be safely managed with lobectomy because of its low malignancy potential, total thyroidectomy appeared to be the best surgical approach in this case due to the existence of parathyroid malignancy. This lowers the risk of further neck surgeries that lead to serious complications in the future.

## Conclusions

The preoperative diagnosis of synchronous existence of parathyroid carcinoma and thyroid neoplasms poses several difficulties because of the rarity of the condition and accordingly requires the constellation of thorough clinical, radiologic, and pathologic investigations. Our experience illuminates the importance of considering parathyroid carcinoma in case of marked hyperparathyroidism and hypercalcemia. The resection of the parathyroid tumor with the ipsilateral thyroid lobe or in combination with total thyroidectomy may sometimes be necessary in case of intraoperative suspicion of malignancy.
